# IL-27 alleviates the bleomycin-induced pulmonary fibrosis by regulating the Th17 cell differentiation

**DOI:** 10.1186/s12890-015-0012-4

**Published:** 2015-02-18

**Authors:** Zhaoxing Dong, Xin Lu, Yanni Yang, Tao Zhang, Yongxia Li, Yanlin Chai, Wen Lei, Changbo Li, Li Ai, Wenlin Tai

**Affiliations:** Department of Respiratory, The 2nd Affiliated Hospital of Kunming Medical University, Kunming, Yunnan China; Department of Hospital Services, The 2nd Affiliated Hospital of Kunming Medical University, Kunming, Yunnan China; Department of Scientific Research and Postgraduate Education, The 2nd Affiliated Hospital of Kunming Medical University, Kunming, Yunnan China; Department of Clinical Laboratory, Yunnan Molecular Diagnostic Center, The 2nd Affiliated Hospital of Kunming Medical University, Dianmian Road, Kunming, Yunnan China 650101

**Keywords:** Interleukin 27, Pulmonary fibrosis, Th17 cells, Animal model, Signaling pathway

## Abstract

**Background:**

Interleukin-27 (IL-27) is a multifunctional cytokine with both pro-inflammatory and immunoregulatory functions. At present, the role of IL-27 in pulmonary fibrosis remains unknown.

**Methods:**

In this study, we observed the expression of IL-27/IL-27R in a mouse model of bleomycin (BLM)-induced pulmonary fibrosis. We verified the role of IL-27 using hematoxylin and eosin as well as Masson’s staining methods and measuring the content of hydroxyproline as well as collagen I and III. We assessed the differentiation of T lymphocytes in the spleen and measured the concentration of cytokines in bronchoalveolar lavage fluid (BALF) and the expression level of relevant proteins in the JAK/STAT and TGF-ß/Smad signaling pathways in lung tissue.

**Results:**

Increased IL-27 expression in BLM-induced pulmonary fibrosis was noted. IL-27 treatment may alleviate pulmonary fibrosis and increase the survival of mice. IL-27 inhibited the development of CD4^+^ IL-17^+^, CD4^+^ IL-4^+^ T, and CD4^+^ Foxp3^+^ cells and the secretion of IL-17, IL-4, IL-6, and TGF-ß. IL-27 induced the production of CD4^+^ IL-10^+^ and CD4^+^ INF-γ^+^ T cells. IL-27 decreased the levels of phosphorylated STAT1, STAT3, STAT5, Smad1, and Smad3 but increased the level of SOCS3.

**Conclusions:**

This study demonstrates that IL-27 potentially attenuates BLM-induced pulmonary fibrosis by regulating Th17 differentiation and cytokine secretion.

## Background

Pulmonary fibrosis is a heterogeneous disease with multiple etiologies. Its primary pathological characteristics include early diffuse fibrosing alveolitis followed by pathologic hyperplasia of fibroblasts and collagen accumulation replacing normal lung tissue [1]. Until recently, its mechanism was not completely understood. Our previous studies explored the potential roles of Th17 lymphocytes and IL-17 in promoting pulmonary fibrosis [2,3]. Various factors regulate Th17 cell differentiation and influence pulmonary fibrosis. Recent evidence suggests that IL-27, including EB13, an IL-12p40 homologous protein, and p28 [4] have unique roles in T cell differentiation. IL-27 inhibits the development of pro-inflammatory Th17 cells by suppressing the expression of the Th17 transcription factor RORγt, thereby preventing the production of interleukin (IL)-17A and IL-17 F in naive T cells [5].

IL-27, an IL-6/IL-12 family cytokine, plays a crucial role in immune regulation in the context of infection and autoimmunity. IL-27 is produced by antigen-presenting cells, such as monocytes, macrophages, and dendritic cells [6,7]. IL-27 also plays an important role in innate and adaptive immunity. In adaptive immunity, IL-27 synergizes with IL-12 to induce IFN-γ production by CD4, CD8 T cells and NK cells [8,9]. In innate immunity, IL-27 promotes the production of IL-1 and TNFα in mast cells as well as IL-8 and IL-12 in monocytes [10]. Kim and colleagues have indicated that the TLR2-mediated production of IL-27 and chemokines by respiratory epithelial cells promotes bleomycin (BLM)-induced pulmonary fibrosis (BIPF) in mice, In addition, BIPF was more severe in IL-17A^−/−^ mice and in TLR2^−/−^ mice treated with an anti–IL-17 monoclonal antibody than in TLR2^−/−^ and wild-type (WT) mice [11]. However, their conclusion regarding IL-17’s action in BIPF is inconsistent with other research [12,13], and they did not explore the direct mechanism of IL-27 in BIPF.

In the present study, we demonstrate the role of IL-27 in BIPF and provide a possible mechanism via immune regulation of the JAK/STAT and TGF-ß/Smad signaling pathways.

## Methods

### Bleomycin induced-pulmonary fibrosis

Male C57/BL mice (aged 7–8 weeks) were purchased from Weitonglihua Company (Beijing). Mice used for experiments were housed in a specific pathogen-free (SPF) room. All procedures were performed in accordance with the Declaration of Helsinki of the World Medical Association. The protocols were also approved by the IRB/Ethics Committee of Kunming Medical University. For the pulmonary fibrosis model, 5 mg/kg BLM (Japan) was dissolved in phosphate-buffered saline (PBS) buffer and administered to the mice intratracheally. On days 3, 7, 14 and 28 following BLM injection, 3 mice were sacrificed, and samples were collected. Either the mouse IL-27 recombinant protein (rmIL-27; 1 μg per mouse for seven days) or anti-mouse IL-27 p28 functional grade purified antibody (200 μg per mouse for one day; R&D System, Minneapolis MN) was injected hypodermically. The mice were sacrificed on days 7 and 28 after BLM or saline administration (3 mice/group were randomly selected for each time point). The mice were divided into four groups: a control group with PBS buffer (n = 6); BLM group (n = 12); BLM + IL-27 group (n = 6) and BLM + IL-27 antibody group (n = 6).

### Tissue preparation and assessment of fibrosis

Whole lung tissues from the mice were dissected and fixed for 1 day in 4 % paraformaldehyde. Then, the samples were dehydrated and embedded in paraffin blocks. Blocks of 6 μm thickness were cut and stained with hematoxylin and eosin (HE) and Masson’s reagent (Shanghai Bogoo Biotechnology. Co., Ltd) for the assessment of pulmonary alveolitis and pulmonary fibrosis, respectively. The histologic severity of the pulmonary alveolitis and pulmonary fibrosis was scored as previously described [14].

### Flow cytometry analysis

Lymphocytes were separated from spleen tissue samples using lymphocyte separation medium (Solarbio Biotec, Beijing). The cells were counted, and 5 × 10^5^ to 1 × 10^6^ cells were harvested in cold PBS. The cells were then resuspended in 100 μl PBS and incubated with 0.5 μg anti-mouse CD3a PE, 0.25 μg anti-mouse CD4 FITC, 0.125 μg anti-mouse IFN gamma PE, 0.25 μg anti-mouse IL-4 FITC, 1 μg anti-mouse/rat Foxp3 FITC, 0.25 μg anti-mouse IL-10 FITC or isotype control antibodies (eBioscience, San Diego, CA). The cells were then washed with cold PBS, and 10^4^ cells per sample were analyzed by flow cytometry (CyFlow, PARTEC).

### Enzyme-linked immunosorbent assay (ELISA)

We collected bronchoalveolar lavage fluid (BALF) and lung tissue samples from the various groups. The hydroxyproline (HYP) content of the lung tissue was measured using an ELISAs. The levels of IL-4, IL-10, IL-6, IL-17, IFN gamma and TGF-β in the BALF were also assessed using ELISAs. All ELISA kits were purchased from CUSBIO Company and used according to the manufacturer’s instructions.

### Quantitative real-time PCR analysis

Total RNA was isolated from lung tissue using TRIzol reagent (TAKARA). IL-27, IL-27R, COL1 and COL3 gene expression was measured using real-time polymerase chain reaction (RT-PCR) performed on a Real-Time PCR System (Applied Biosystems, ABI Prism 7300, American) instrument with SYBRGreen (TAKARA), starting with 1 ng of reverse-transcribed total RNA. PCR was performed under the following conditions: 95°C for 5 min followed by 30 cycles of 95°C for 30 s and 60°C for 30 s. The following oligonucleotide primers specific for the mouse genes and β-actin were used: IL-27 p28, 5′-CTT CCT CGC TAC CAC ACT TCG-3′ (sense) and 5′-CCT CTT CCT CCT TGT CCT CCT-3′ (antisense); IL-27R, 5′-GCT CTG CCC TGG TTT CTG TC-3′ (sense) and 5′-CTC CTT GAT GTA AGG TTG CCC-3′ (antisense); COL1, 5′-GGG TGA GAC AGG CGA ACA AG-3′ (sense) and 5′-AAC CAG CAG AGC CAG GGG-3′ (antisense); COL3, 5′-TGC CCA CAG CCT TCT ACA CC-3′ (sense) and 5′-GCC ACC CAT TCC TCC CAC-3′ (antisense); and β-actin, 5′-CGT GCG TGA CAT CAA AGA GAA G-3′ (sense) and 5′-CCA AGA AGG AAG GCT GGA AAA-3′ (antisense). The amplification efficiency between the target and the reference control (GAPDH) was compared to use the delta delta Ct (ΔΔCt) calculation.

### Western blot analysis

Lung tissues were lysed in Tissue Protein Lysis Solution (CWBIOTECH, Beijing). Protein from each sample was loaded into a 10 % sodium dodecyl sulfate (SDS)-polyacrylamide electrophoresis gel and transferred to a nitrocellulose membrane (Amersham Biosciences, Piscataway, NJ). The blocked membrane was then incubated with various antibodies. Antibodies against IL-27 (C-8), COL1A2 (M-19), p-Stat1 (Tyr 701), p-Stat5 (Tyr 694/Tyr 699), p-Smad3 (Ser 208), TGF-βR1 (V-22), p-Stat3 (B-7), and p-Smad1 (Ser 465) were purchased from Santa Cruz Biotechnology. Antibodies against SOCS3, SMAD1, STAT3, STAT5, STAT1, SMAD3, and β-actin were purchased from Proteintech. The immunoreactive bands were visualized with an enhanced chemiluminescent (ECL) reagent (Beyotime). The signal intensity of the bands was quantified using Image J software.

### Statistical analysis

Data are expressed as the mean ± SD or median (range). Statistical significance was determined using analysis of variance (ANOVA), and the difference between two groups was determined using the Student-Newman-Keuls test. All tests were performed using SPSS 17.0 software. A p-value < 0.05 was considered statistically significant.

## Results

### IL-27/IL-27R expression increases in bleomycin-induced pulmonary fibrosis

**Figure 1:**
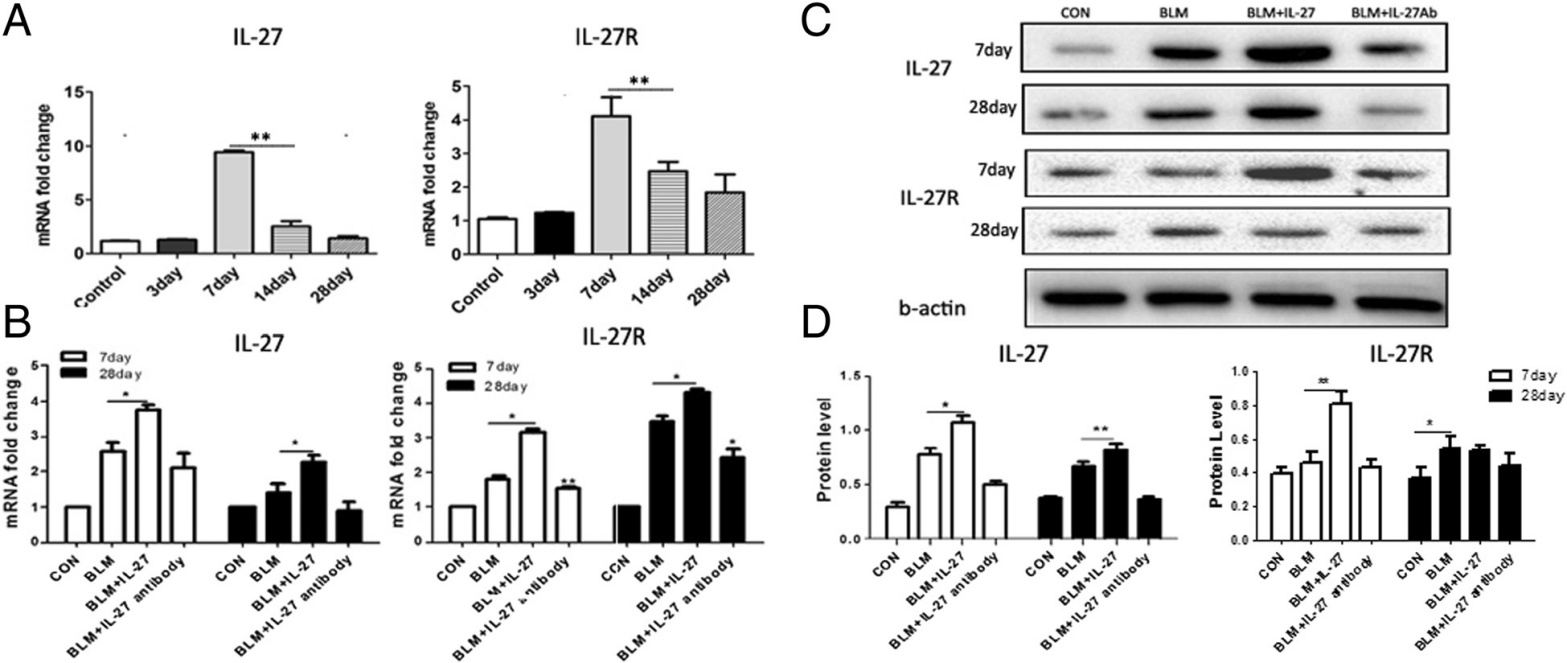
**IL-27/IL-27R expression increases in bleomycin-induced pulmonary fibrosis.** **A**. Real-time PCR for IL-27 and IL-27R, at days 3, 7, 14, and 28 in the BLM and control groups. The expression of the CT values for real-time PCR were normalized by 2^-∆∆ct^. **B**. IL-27 and IL-27R mRNA expression in the different groups. **C**, **D**. Western blot analysis; band intensities were measured using Image J. For each group, n = 3. Data are expressed as the mean ± SEM. *p < 0.05, **p < 0.01.

To explore the role of IL-27 in the pathogenesis of pulmonary fibrosis, we assessed IL-27/IL-27R mRNA expression in BIPF using quantitative RT-PCR. IL-27/IL-27R expression was the highest on day 7 of BIPF(1A, B). We then treated these mice with either IL-27 or an IL-27 antibody and observed the level of IL-27/IL-27R 7 and 28 days later (Figure 1B, C, D). Increased IL-27 was noted in the BLM + IL-27 group compared with the other groups. The level of IL-27 at 28 days was increased compared with the levels observed at 7 days for all groups except the control group. These results indicate that IL-27 may be involved in the formation of BIPF.

### IL-27 attenuates bleomycin-induced pulmonary fibrosis

**Figure 2:**
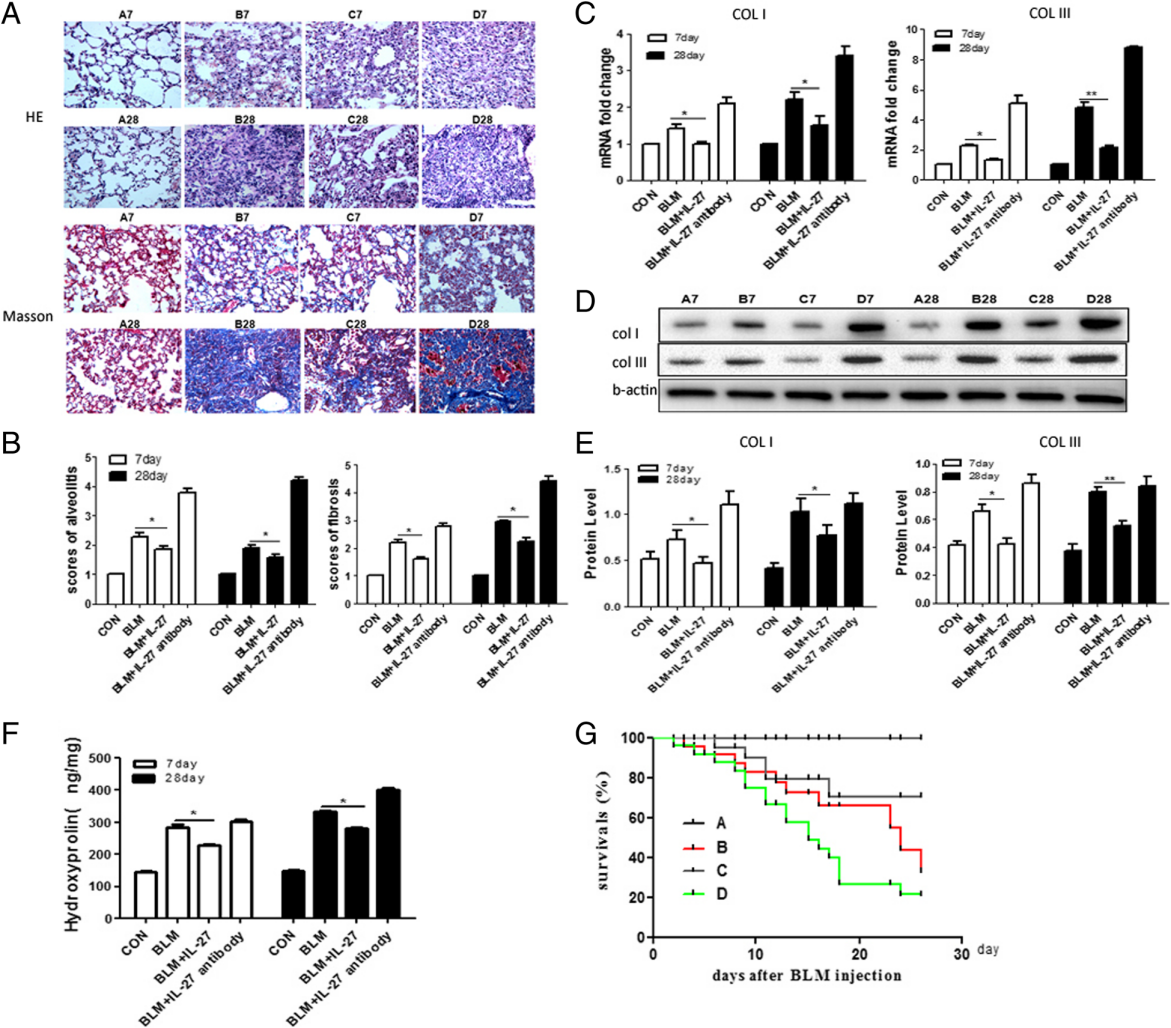
**Exogenous IL-27 treatment can suppress BLM-induced pulmonary fibrosis.** **A**. Hematoxylin and eosin (HE) staining (100x) and Masson’s trichrome staining (100x) depicting the effects of exogenous IL-27 application on lung histological changes at days 7 and 28 in various treated mice. **B**. The alveolitis and fibrosis scores for the lungs of mice in the different groups at days 7 and 28. Values are means ± SEM (n = 5). **C**, **D**, **E**. Real-time PCR and Western blot analysis for COL1 and COL3, respectively, in the different groups. CT values for the real-time PCR were normalized by 2^-∆∆ct^. Western band intensities were measured using Image J software. For each group, n = 3. **F**. The lung hydroxyproline content at days 7 and 28 for mice in the various treatment groups. Hydroxyproline content was measured using an ELISA, n = 5. **G**. Survival rate of mice with BLM-induced pulmonary fibrosis. The percentage of survival over time is presented, n = 5. Data are expressed as means ± SEM. *p < 0.05, **p < 0.01. A: control, B: BLM group, C: BLM + IL-27 group, D: BLM + IL-27 antibody group.

To verify the role of IL-27 in BIPF, the mice were divided into four groups: a control group, BLM group, BLM + IL-27 group, and BLM + IL-27 antibody group. HE staining revealed a disordered lung tissue structure; the pulmonary interalveolar septa were thickened and infiltrated by inflammatory cells, and a large number of alveoli were collapsed. These observations were consolidated in the BLM group and most severe in the BLM + IL-27 antibody group, whereas injected IL-27 alleviated the degree of alveolitis. Based on Masson’s staining, wherein blue stain represents the fibroblasts and collagen matrix, a similar trend was observed (Figure 2A, B).

In different tissues, hydroxyproline can act as a primary indicator of collagen metabolism. To more accurately illustrate the severity of pulmonary fibrosis, we measured the hydroxyproline content of the lung tissue samples on days 7 and 28 in the four groups. We found that the hydroxyproline content in the BLM group was greater than that of the BLM + IL-27 group, whereas the hydroxyproline content of the lung was significantly increased in the group administered the IL-27 antibody (Figure 2F). At the same time, we also measured the levels of collagen I and III; the results were similar to the hydroxyproline results (Figure 2C, D, E).

Over the course of the experiment, we noted the survival rate of the mice and found that the BLM and BLM + IL-27 antibody groups exhibited an increased mortality rate compared with the other groups, with the BLM + IL-27 antibody group exhibiting the highest survival rate. Surprisingly, the survival of mice in the BLM + IL-27 group was the highest and most comparable to the control group (Figure 2G). From the above results, we hypothesized that IL-27 inhibits BIPF.

### IL-27 regulates the differentiation of T lymphocytes

**Figure 3:**
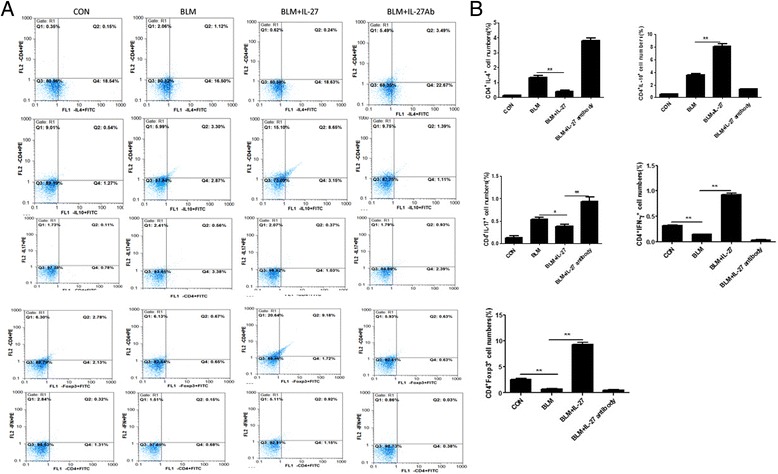
**The effect of IL-27 treatment on T lymphocyte differentiation in the spleen after 28 days.** **A**. Representative figures from flow cytometry analyses; CD4^+^ IL-4^+^, CD4^+^ IL-10^+^, CD4^+^ IL-17^+^, CD4^+^ Foxp3^+^ and CD4^+^ IFN-γ^+^ cells from the spleen were analyzed on day 28. **B**. Analyzed data. Values are expressed as means ± SEM (n = 3). *p < 0.05, **p < 0.01.

To explore the mechanisms by which IL-27 inhibits BIPF, we evaluated T lymphocyte differentiation in spleen tissues at day 28. Using intracellular fluorescence staining as assessed by flow cytometry, we measured the CD4+ cells also expressing Foxp3, IL-10, IL-17, IFN-γ, and IL-4. Compared with the control group, the BLM and BLM + IL-27 antibody groups exhibited increased numbers of IL-4-producing CD4+ cells, IL-10-producing CD4+ cells and IL-17-producing CD4+ cells as well as a reduced number of IFN-γ − producing CD4+ cells and Foxp3-producing CD4+ cells. However, after the IL-27 treatment, these expression patterns were reversed with the exception of the IL-10-producing CD4+ cells (Figure 3A, B). The above findings suggest that IL-27 inhibits pulmonary fibrosis by regulating T lymphocyte differentiation in the spleen.

### IL-27 influences IL-17, IL-6, IL-4, IL-10, IFN-γ, and TGF-β secretion

**Figure 4:**
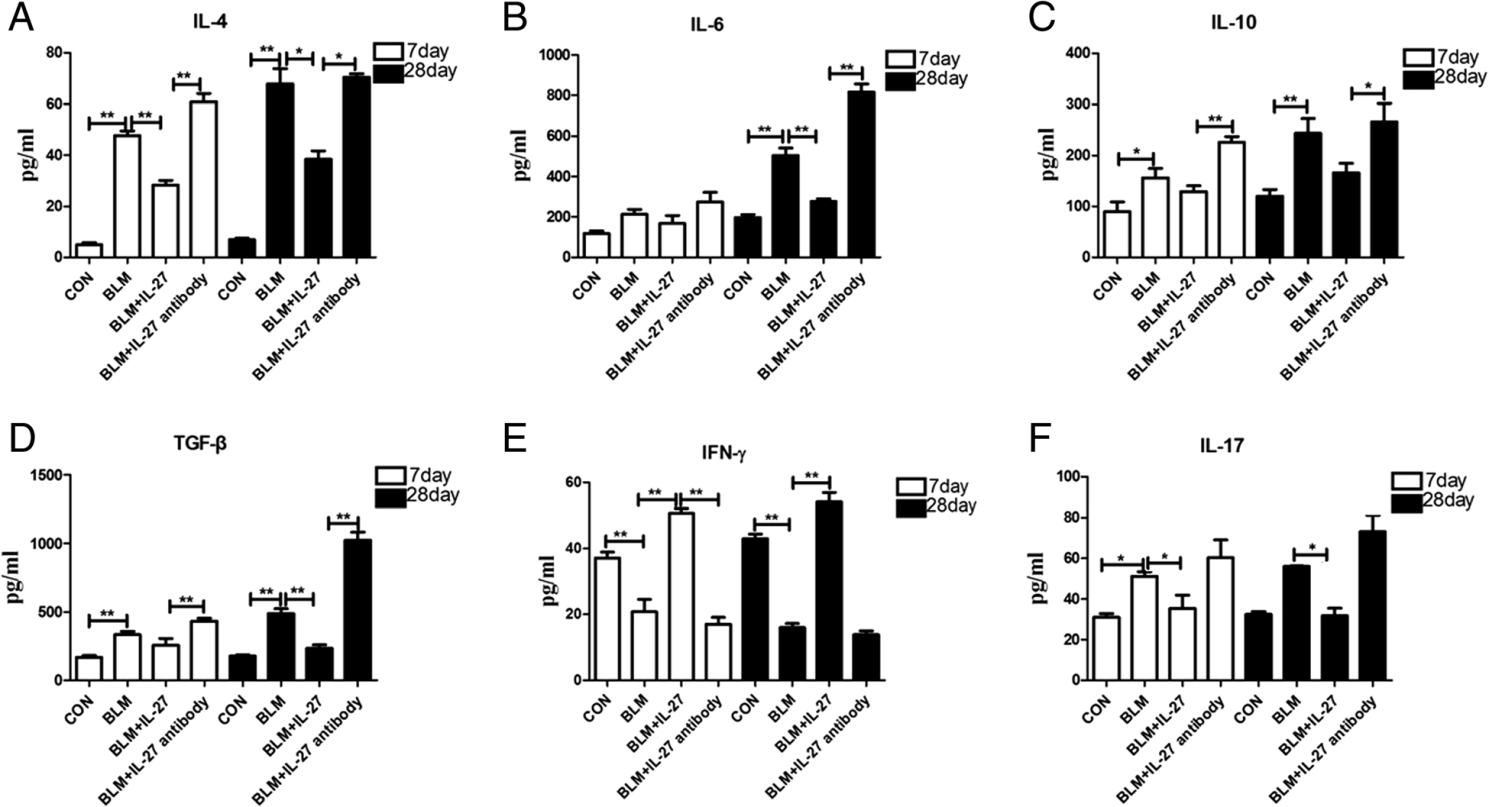
**The concentration of cytokines in the BALF of the different groups on days 7 and 28.** The concentration (in pg/ml) of **(A)** IL-4; **(B)** IL-6; **(C)** IL-10 ; **(D)** TGF-β; **(E)** IFN-γ; and **(F)** IL-17. Data are expressed as means ± SEM (n = 3). *p < 0.05, **p < 0.01.

We also measured the concentrations of IL-4, IL-6, IL-10, IL-17, IFN-γ and TGF-β in the BALF of mice at days 7 and 28 using an ELISA. In the BIPF model, IL-4, IL-10, IL-17 and TGF-β were significantly increased compared with the control group at days 7 and 28. IL-6 was also increased at day 28. Conversely, IFN-γ was significantly decreased at days 7 and 28. These trends were exacerbated following IL-27 antibody treatment. Conversely, IL-27 treatment reversed these trends (Figure 4A-F). Therefore, we speculate that IL-27 may inhibit pulmonary fibrosis by influencing the expression of inflammatory factors.

### IL-27 regulates the JAK/STAT, TGF-ß/Smad signaling pathway

To investigate the mechanism by which IL-27 acts in pulmonary fibrosis, we determined JAK, STAT1, STAT3, STAT5, and SOCS3 protein levels as well as STAT1, STAT3, and STAT5 phosphorylation levels using Western blot analysis. These results indicated that although the total amounts of STAT1, STAT3, and STAT5 did not significantly differ among the groups, BIPF increased the levels of phosphorylated STAT1, STAT3, and STAT5. IL-27 treatment decreased the expression of these phosphorylated proteins. Conversely, the expression of SOCS3 was increased in the BLM + IL-27 group and decreased in the BLM group compared with the control group.

**Figure 5:**
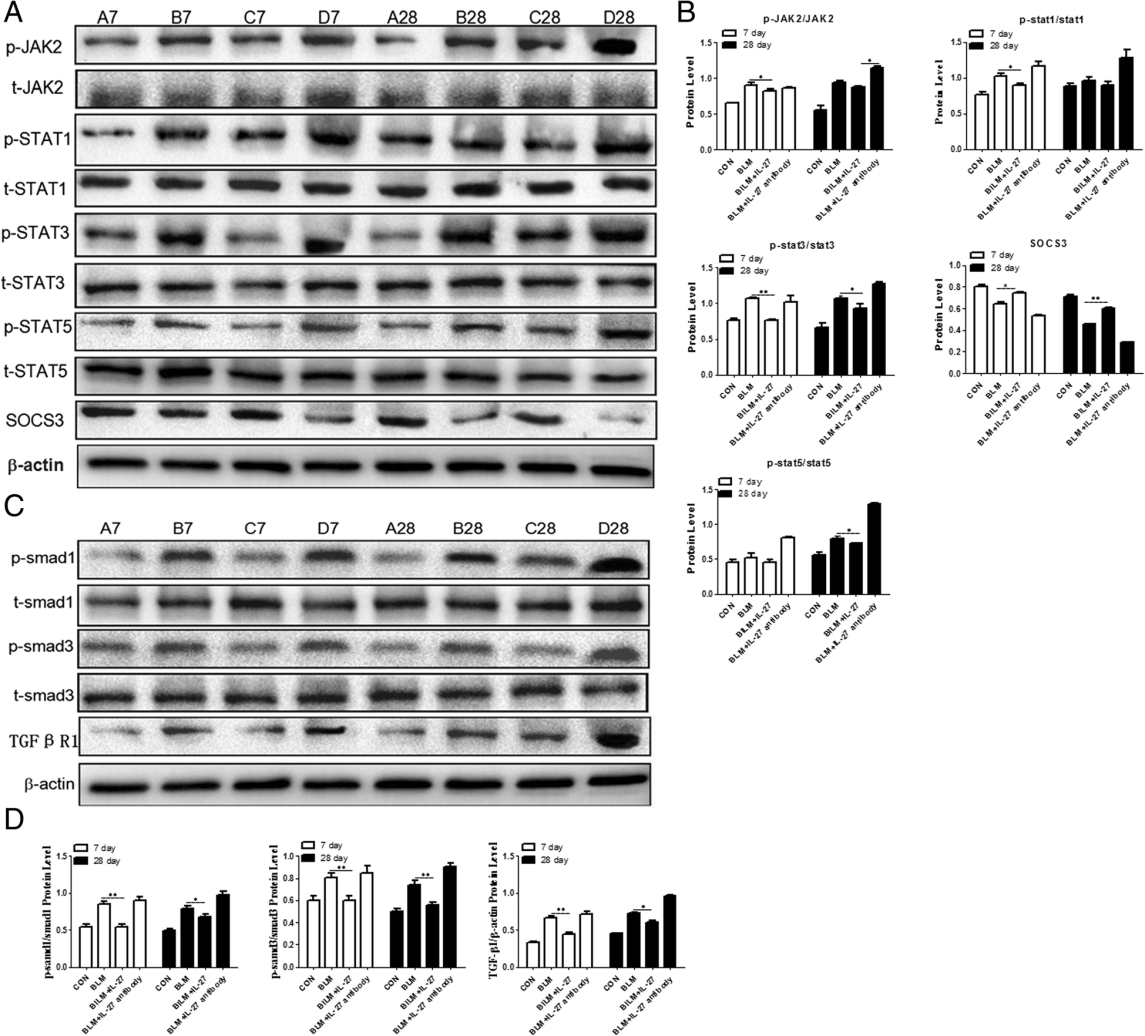
**The expression of JAK/STAT and TGF-β/Smad signaling pathway-related proteins in the lung tissues of the different groups on days 7 and 28. A**. Western blots of JAK/STAT signaling pathway-related proteins: JAK, STAT1, STAT3, STAT5, SOCS3, and phosphorylated STAT1, STAT3, and STAT5. **B**. A histogram of the relative gray values shown in **A**. **C**. Western blots for the TGF-β/Smad signaling pathway-related proteins: Smad1, Smad3, TGF-βR1 and phosphorylated Smad1 and Smad3. **D.** A histogram of the relative gray values shown in **C**. Data are expressed as means ± SEM (n = 3). *p < 0.05, **p < 0.01.

Previous ELISA results for TGF-β have suggested that IL-27 inhibits TGF-β expression. To further assess the relationship between IL-27 and the TGF-β signaling pathway, we determined TGF-βR1, Smad1, and Smad3protein levels. We found that the levels of TGF-βR1 and phosphorylated Smad1 and Smad3 were increased in the BLM group compared with the control group. However, IL-27 treatment resulted in decreased levels of these proteins at days 7 and 28 (Figure 5A-D). These results imply that IL-27 also inhibits activation of the JAK/ STAT and TGF-β/Smad signaling pathways in BIPF.

## Discussion

In this study, our data demonstrate that IL-27 possesses variable functions in pulmonary fibrosis. IL-27 may play a role in diffuse alveolitis and fibroblast hyperplasia. To explore the role of IL-27, we treated mice with BIPF with either recombinant IL-27 protein or IL-27 antibodies. The results verified that IL-27 alleviates pulmonary fibrosis. IL-27 also enhances the survival of mice with pulmonary fibrosis. Kim et al. proposed that respiratory epithelial cells produce IL-27 and chemokines in a TLR2-dependent manner to promote BIPF [11]. This result is inconsistent with our findings. The main objective of their experiment was to identify the respiratory epithelial cells, rather than the immune cells, that play a critical role in TLR2-mediated regulation of BLM-induced tissue injury, which may explain the discrepancies with our study.

IL-27 is a double-edged sword involved in offense and defense [15]. IL-27 is a key cytokine that drives naive cells into the Th1 subset during the initial step of differentiation [16,17]. IL-27 also has an immunosuppressive function that suppresses the production of inflammatory cytokines. Several investigations have indicated that this cytokine promotes the secretion of pro-inflammatory cytokines by human monocytes [18] and has broad inhibitory effects on Th1, Th2, Th17, and T-regulatory cells [19-21]. Here, we explored the mechanism of IL-27 action in BIPF. Our results indicate that IL-27 inhibited the differentiation of Th1, Tr1, and Th17 and promoted Treg and Th2 expression. IL-10 is most commonly recognized as an anti-inflammatory cytokine possessing immunosuppressive effects that are necessary for the regulated resolution of pro-inflammation mechanisms. Various research indicates that IL-10 over-expression induces fibrosis partly through fibrocyte recruitment and M2 macrophage activation, which likely involves a CCL2/CCR2 axis [22]. In our study, although we did not directly demonstrate the role of IL-10, we also observed that IL-10 levels were elevated in BIPF at 7 and 28 days. IL-27 treatment decreased IL-10 expression. Two important classes of Tregs within the CD4^+^ subset are the CD4^+^ CD25^+^ Foxp3^+^ Tregs and the T regulatory type 1 (Tr1) cells [23]. Tr1 cells are CD4^+^ T lymphocytes defined by their ability to produce IL-10 and suppress helper T cells. We found that the expression of CD4^+^ CD25^+^ Foxp3^+^ Tregs was inhibited, whereas the number of CD4^+^ IL-10^+^ T cells was increased in BIPF. IL-27 treatment changed these results.

In the BIPF model, we observed elevated levels of TGF- β and IL-6, which is consistent with other reports [24]. In mice, TGF-β and IL-6 or TGF-β and IL-21 induce the differentiation of naive T cells toward the Th17 phenotype [25]. The role of Th17 cells and their secretion of IL-17 (IL-17A) in pulmonary fibrosis has been previously demonstrated by our group as well as others [2,3,12,26]. In this experiment, our results indicate that IL-27 attenuates pulmonary fibrosis by regulating T cell differentiation and the secretion of IL-17 and other cytokines.

Research has demonstrated that the JAK/STAT signaling pathway is involved in pulmonary fibrosis [27]. IL-27 also activates the STAT1, STAT3, and STAT6 transcription factors. The activation of STAT1 and STAT3 by IL-27 has been described in T cells, monocytes, and keratinocytes [28,29]. However, this study is the first to report that IL-27 suppresses STAT1 and STAT3 phosphorylation in BLM-induced lung tissue. A detailed mechanism underlying IL-27’s effect on the JAK/STAT3 signaling pathway remains unclear. Dysregulated SOCS3 activity may STAT3 signal transduction in fibroblasts/myofibroblasts in idiopathic pulmonary fibrosis (IPF). The activity of SOCS3 was suppressed in BIPF, an effect that was reversed by IL-27. The TGF-β/SMAD signaling pathway could also mediate fibrosis via the mechanism of the epithelial-mesenchymal transition (EMT) [30]. Therefore, we also identified whether IL-27 influences the TGF-β signaling pathway in BIPF, but a detailed mechanism will require further investigation.

## Conclusion

In summary, we verified that IL-27 is involved in BIPF. In addition, IL-27 potentially inhibits the pulmonary fibrosis by regulating T cell differentiation and the secretion of IL-17 and other cytokines and suppressing the JAK/STAT and TGFβ1/SMAD signaling pathways. These results have important implications for the treatment of pulmonary fibrosis.

## Abbreviations

BLM: Bleomycin
mRNA: messenger RNA
BLAF: Bronchoalveolar lavage fluid
TGF-β: Transform growth factor β
PF: Pulmonary fibrosis
CON: Control
HE: Hematoxylin and eosin
MDC: Monodansylcadaverine
HYP: Hydroxyproline
ELISA: Enzyme-linked immunosorbent assay
